# Pan-cancer and single-cell analysis reveal THRAP3 as a prognostic and immunological biomarker for multiple cancer types

**DOI:** 10.3389/fgene.2024.1277541

**Published:** 2024-01-25

**Authors:** Ye-Peng Wang, Chao Ma, Xue-Kun Yang, Nan Zhang, Zhi-Gang Sun

**Affiliations:** ^1^ Department of Neurosurgery, Central Hospital Affiliated to Shandong First Medical University, Jinan, Shandong, China; ^2^ Department of Thoracic Surgery, Central Hospital Affiliated to Shandong First Medical University, Jinan, Shandong, China; ^3^ Department of Neurology, Central Hospital Affiliated to Shandong First Medical University, Jinan, Shandong, China; ^4^ Breast Center, Central Hospital Affiliated to Shandong First Medical University, Jinan, Shandong, China

**Keywords:** biomarker, pan-cancer, immune infiltration, prognosis, single cell

## Abstract

**Background:** Thyroid hormone receptor-associated protein 3 (THRAP3) is of great significance in DNA damage response, pre-mRNA processing, and nuclear export. However, the biological activities of THRAP3 in pan-cancer remain unexplored. We aimed to conduct a comprehensive analysis of THRAP3 and validate its expression levels in lung cancer.

**Methods:** A pan-cancer analysis was conducted to study the correlation of THRAP3 expression with clinical outcome and the tumor microenvironment based on the available bioinformatics databases. The protein levels of THRAP3 were explored in lung cancer by immunohistochemistry (IHC) analysis. Single-cell sequencing (ScRNA-seq) analysis was employed to investigate the proportions of each cell type in lung adenocarcinoma (LUAD) and adjacent normal tissues, along with the expression levels of THRAP3 within each cell type.

**Results:** THRAP3 is upregulated in multiple cancer types but exhibits low expression in lung squamous cell carcinoma (LUSC). immunohistochemistry results showed that THRAP3 is a lowly expression in LUAD and LUSC. THRAP3 elevation had a poor prognosis in kidney renal clear cell carcinoma and a prolonged survival time in kidney chromophobe, brain lower-grade glioma and skin cutaneous melanoma, as indicated by the KM curve. Single-cell analysis confirmed that the proportions of T/B cells, macrophages, and fibroblasts were significantly elevated in LUAD tissues, and THRAP3 is specifically overexpressed in mast cells.

**Conclusion:** Our findings uncover that THRAP3 is a promising prognostic biomarker and immunotherapeutic target in multiple cancers, but in LUAD and LUSC, it may be a protective gene.

## Introduction

Cancer poses a grave risk to human life and health, and lung cancer has the greatest fatality rate (Thai et al., 2021; Ma et al., 2022a; Ma et al., 2022b; Siegel et al., 2023). There is currently no effective treatment for cancer. Recent research indicates that immunotherapy, as exemplified by immune checkpoint inhibitors, has demonstrated more significant potential in treating lung cancer and other cancers (Doroshow et al., 2021; Passaro et al., 2022; Ma et al., 2023a; Ma et al., 2023b). Therefore, we aimed to identify possible biomarkers and therapeutic targets for tumors by conducting comprehensive analysis, based on the Cancer Genome Atlas (TCGA) and Gene Expression Omnibus (GEO) datasets.

THRAP3 is a nuclear receptor coactivation factor that interacts with multiple nuclear receptors in a ligand-dependent manner. THRAP3 is structurally located in chromosome 1p34. This transcript encoded 955 amino acids with molecular masses of 150 kDa. Functionally, THRAP3 is involved in nuclear export, DNA damage repair, and the maintenance of genomic stability (Beli et al., 2012; Voh et al., 2017).

The study by Li et al. found that THRAP3 is significantly correlated with the infiltration levels of immune cells and immune-related genes in kidney renal clear cell carcinoma (KIRC) (Li et al., 2022). THRAP3 was reported to be a tumor suppressor in BRAF-mutated colorectal cancer (Ma et al., 2022c). Mechanistically, nuclear PD-L1 interacts with THRAP3 to increase BUB1 expression, leading to cell proliferation. In cancer cells, THRAP3 facilitates cell growth by inducing R-loop resolution (Kang et al., 2021). In addition, phosphorylated THRAP3 is involved in tumor progression in the androgen-independent prostate (Ino et al., 2016).

Several studies have implicated THRAP3 in the development of certain cancers. However, there is no comprehensive analysis of the biological role of THRAP3 expression in pan-cancer. In order to investigate the expression level, prognostic significance, molecular function, signaling pathways, and immune infiltration pattern of THRAP3 in 33 kinds of cancer, we conducted a systematic bioinformatics analysis and experimental validation in LUAD.

## Methods

### Data preprocessing and differential expression analysis

RNA-seq and clinical information for 33 cancers were downloaded from the TCGA database. On the UCSC Xena official website (https://xenabrowser.net/datapages/), we obtained the expression profiles of 1,076 tumor cell lines from the CCLE database and 7,862 normal tissues from the Genotype-Tissue Expression (GTEx) database. All RNA-seq data were log2 transformed.

The differential analysis of THRAP3 expression in pan-cancer was carried out based on R Studio (version 4.2.1). *p* < 0.05 was deemed significant.

### Processing of scRNA-seq data

For our study, we chose the scRNA-seq dataset GSE149614 from the GEO database. The dataset contains single-cell RNA sequencing data from 2 LUAD tissues and 2 adjacent normal tissues. We employed the ‘Seurat’ R package to address batch effects among samples (Butler et al., 2018). Cells with a gene count between 100 and 7,500 and a mitochondrial gene proportion below 30% were included in the study, while cells deemed of low quality were excluded from the dataset. We standardized the data using the “Normalize Data" function and employed the “Find Variable Features” function to identify genes with specific expression patterns. The “RunPCA" function was applied for clustering and UMAP visualization dimension reduction on the dataset (Narayan et al., 2021). The “FindMarkers" function was used to analyze DEGs in various cell subtypes. We identified genes specific to each cell cluster and manually annotated cells using the Cellmarker (Hu et al., 2023) and PanglaoDB databases (Franzén et al., 2019). The “DotPlot” function from the “Seurat” package was utilized to create dot plots depicting the expression levels of THRAP3 across cell subtypes. The “FeaturePlot” function was employed to illustrate the expression distribution of each gene in the clustering plot.

### Relationships between THRAP3 and prognosis

On the basis of TCGA data, the link between THRAP3 expression and pathological stage and age was investigated. Using the R packages “survival,” “survminer,” and “forestplot,” a Cox risk regression model was employed to investigate the relationship between THRAP3 expression and survival. To examine the role of THRAP3 in prognosis, the association between THRAP3 expression and over survival (OS), disease specific survival (DSS), disease-free interval (DFI), and progression-free interval (PFI) was studied (Ding et al., 2022; He et al., 2022; Wei et al., 2022). Utilizing the KM curve and the log-rank test, survival studies for each tumor were conducted.

### IHC analysis

Human tissue microarrays of LUAD, and LUSC (R101Lu01; Wuhan Boster Biotechnology, Wuhan, China) were purchased. The clinical characteristics of 30 paired LUSC and LUAD specimens were obtained from the company’s websites. With an anti-THRAP3 (PU300969, 1:500) antibody, immunohistochemistry (IHC) was performed. The chips were scanned using a Leica APERIO VERSA 8 tissue Microarray Scanner. Imagescope software automatically identifies dark brown as strongly positive, brown yellow as moderately positive, light yellow as weakly positive, and blue nuclei as negative on tissue sections and analyzes its area (in pixels), the percentage of positives.

### Relationship between THRAP3 expression and TME

TME is a local homeostatic environment comprised of tumor cells, stromal cells, immune cells, extracellular matrix, and cytokines that develop alongside a tumor and provide the essential material basis for tumor-related malignant phenotypes to develop (Pitt et al., 2016; Xiao and Yu, 2021; Hinshaw and Shevde, 2019).

StromalScore, ImmuneScore and ESTIMATEScore for each cancer were evaluated based on the R package “ESTIAMTE (Yoshihara et al., 2013)." An analysis of the connection between THRAP3 and tumor immunity was conducted using the Pearson method. All results were depicted using the lollipop plot.

### Enrichment analysis

Functional enrichment analysis was utilized to study the biological process of THRAP3 expression, and pathway enrichment analysis was used to explore cancer-related pathways of THRAP3 expression in various cancers. As a differential analysis at pathway levels, Gene Set Variation Analysis (GSVA) was used to investigate the difference in pathways between high- and low-THRAP3 subgroups (Hänzelmann et al., 2013). We carried out Gene Set Enrichment Analysis (GSEA) (Subramanian et al., 2005) and GSVA based on the R packages “ReactomePA,” “org.Hs.e.g.,.db,” “clusterProfiler," “enrichplot,” and “GSEABase.” The GSEA and GSVA gene sets were downloaded from the MSigDB official website (Castanza et al., 2023).

### Genetic alteration analysis and drug sensitivity analysis

Analysis of the alteration frequency of THRAP3 in pan-cancer was performed based on the cBioPortal database (Cerami et al., 2012). Drug sensitivity analysis was conducted to explore the correlation between THRAP3 expression and drugs in the CellMiner database (Luna et al., 2021).

### Statistical analysis

We performed differential analysis of THRAP3 expression using the Wilcoxon test. The correlation analysis was evaluated with Pearson’s or Spearman’s methods. The analyses were conducted with R software (version 4.2.1), and a *p*-value <0.05 was deemed statistically significant.

## Results

### Expression of THTAP3 in various cancers

**FIGURE 1:**
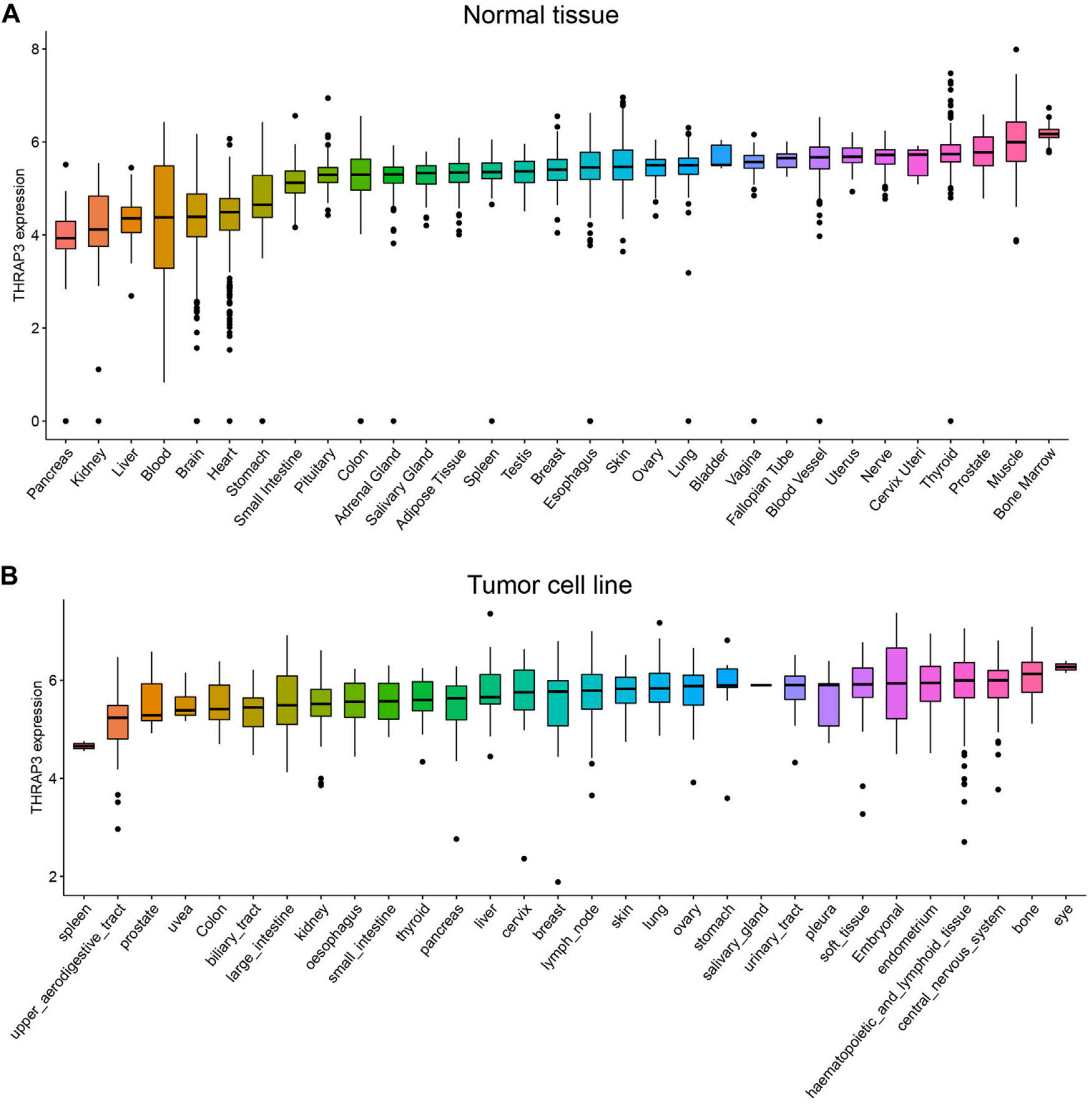
Comparison of THRAP3 expression levels in pan-cancer. **(A)** THRAP3 expression in normal tissues. **(B)** THRAP3 expression in tumor cell lines.

**FIGURE 2:**
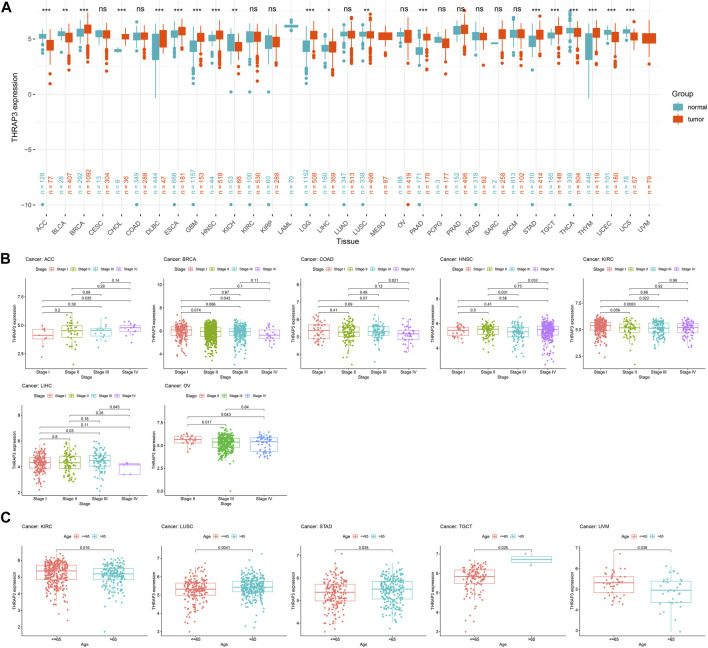
Differential analysis of THRAP3 expression in various cancers. **(A)** The difference in THRAP3 expression between tumor tissues and matched-normal tissues. **(B)** Association between THRAP3 expression and tumor stage. **(C)** Association between THRAP3 expression and age. *p*-value <0.05 was considered statistically significant. * *p*-value <0.05, ** *p*-value <0.01, and *** *p*-value <0.001. “ns” indicated no significance.

THRAP3 expression levels in normal tissues are depicted in Figure 1A using the GTEx database. The highest levels of THRAP3 expression are found in bone marrow, while the lowest levels are found in pancreatic tissue. Based on the CCLE data, THRAP3 expression levels in distinct tumor cell lines were measured and ranked from high to low (Figure 1B). THRAP3 is highly expressed in 12 tumors and lowly expressed in 7 tumors, according to differential analysis in 33 types of cancer (Figure 2A).

### Relationship between THRAP3 and prognosis

We analyzed the differential expression of THRAP3 at various stages for each tumor. THRAP3 expression was found to be substantially linked with stage in 7 malignancies, including adrenocortical carcinoma (ACC), breast invasive carcinoma (BRCA), colon adenocarcinoma (COAD), head and neck squamous cell carcinoma (HNSC), KIRC, liver hepatocellular carcinoma (LIHC), and ovarian serous cystadenocarcinoma (OV). Among BRCA, HNSC, KIRC, and OV, THRAP3 expression in the early stage is higher than in the late stage. While in ACC, THRAP3 expression in late stages is higher than in early stages (Figure 2B).

Furthermore, our findings revealed that THRAP3 expression is closely related to age. In KIRC and uveal melanoma (UVM), THRAP3 expression in patients aged ≤ 65 years is higher than that in patients aged >65 years. In LUSC, stomach adenocarcinoma (STAD), testicular germ cell tumors (TGCT), THRAP3 expression in patients aged >65 years is higher than that in patients aged ≤ 65 (Figure 2C).

**FIGURE 3:**
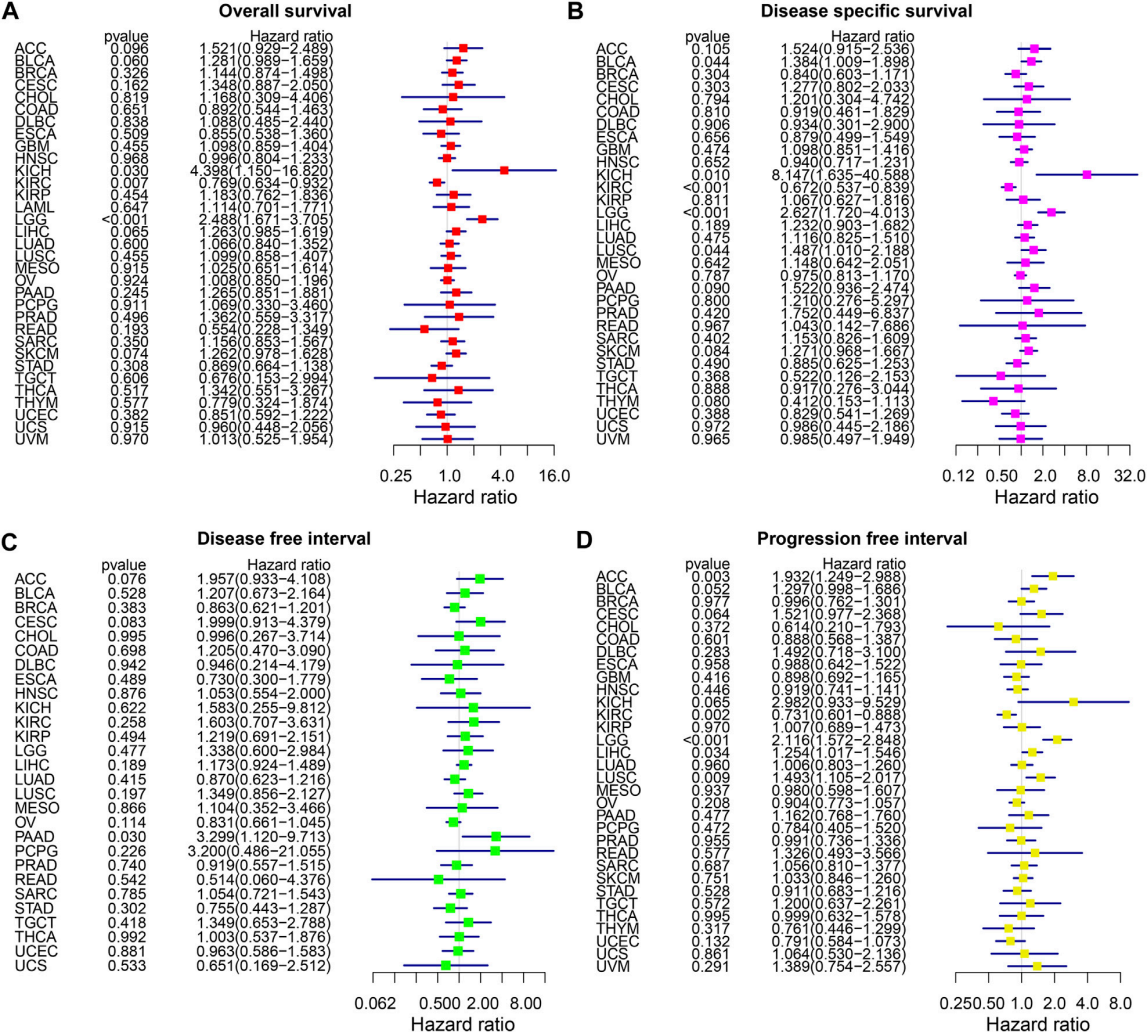
Forest plot of association of THRAP3 expression and OS **(A)**, DSS **(B)**, DFI **(C)**, PFI **(D)** in various cancers.

The forest plot of the OS analysis suggested that THRAP3 is a high-risk gene in KICH and LGG, but a protective gene in KIRC (Figure 3A). THRAP3 expression was positively associated with shorter DSS in BLCA, KICH, LGG, and LUSC and negatively associated with longer DSS in KIRC (Figure 3B). Figure 3C illustrates that THRAP3 is predicted to be a high-risk factor in PAAD. PFI analysis indicates that ACC, LGG, LIHC, and LUSC patients with THRAP3 overexpression had worse PFI, while KIRC patients with low expression had better PFI (Figure 3D).

**FIGURE 4:**
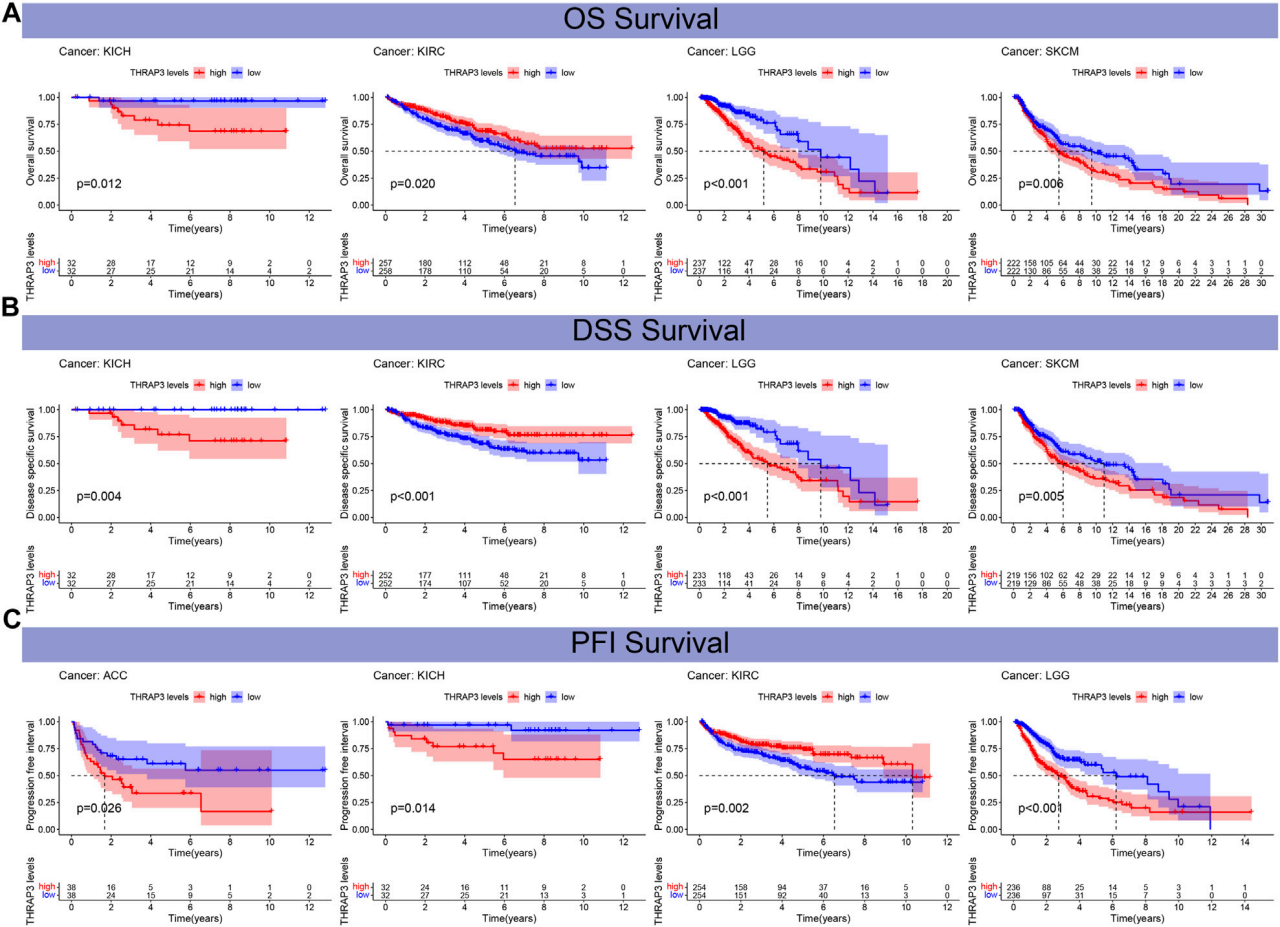
The relationship between THRAP3 and prognosis. **(A)** Kaplan-Meier analysis of the association between THRAP3 expression and OS. **(B)** Kaplan-Meier analysis of the association between THRAP3 expression and DSS. **(C)** Kaplan-Meier analysis of the association between THRAP3 expression and PFI.

Figures 4A, B revealed that THRAP3 overexpression has a longer OS and DSS in patients with KIRC, while in KICH, LGG, SKCM, THRAP3 overexpression has a poor OS and DSS. KM analysis of PFI data showed that THRAP3 elevation was positively correlated with worse PFI in patients with ACC, KICH, and LGG, and it has a longer PFI in KIRC (Figure 4C).

### Relationship between THRAP3 expression and TME

**FIGURE 5:**
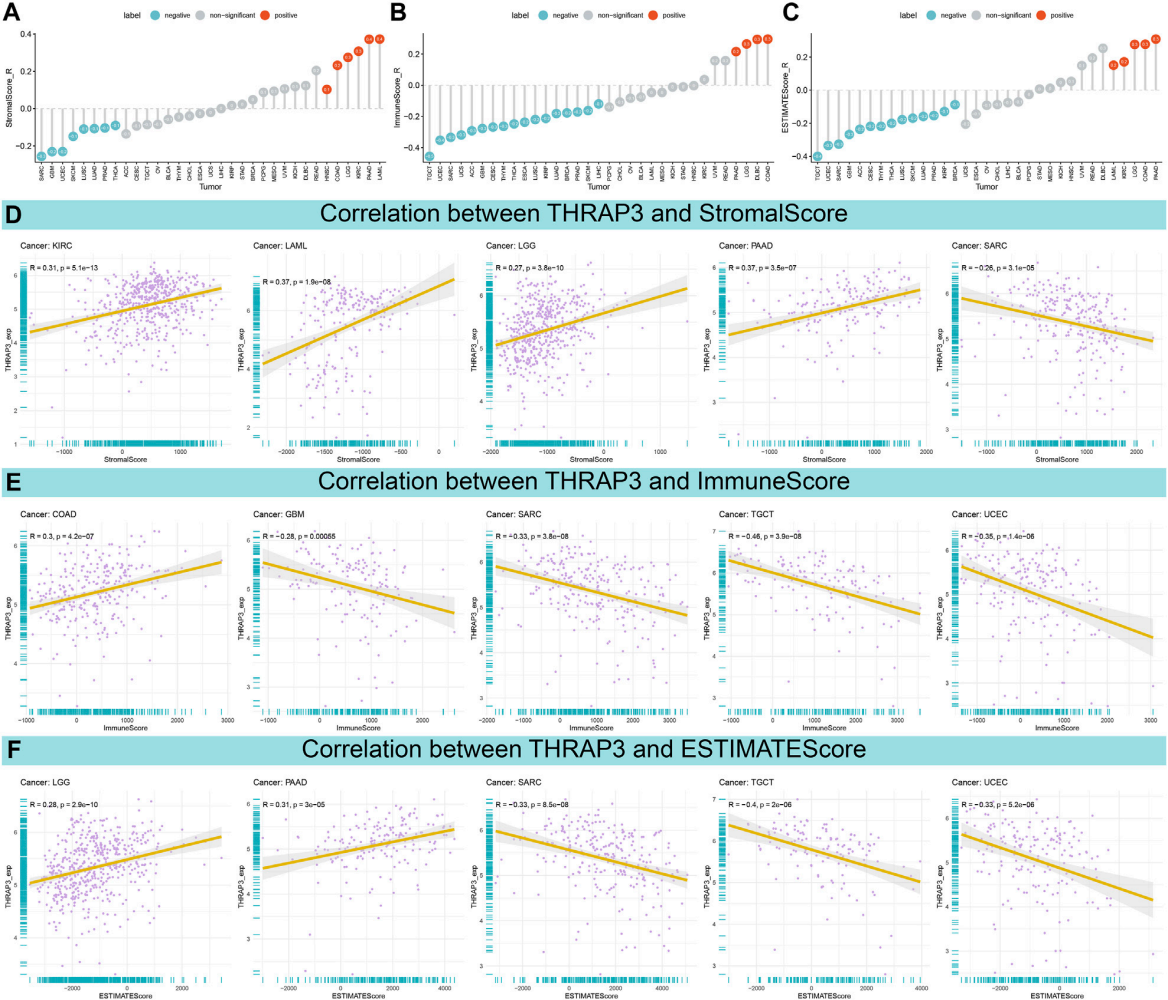
Association between THRAP3 expression and TME. **(A–C)** Lollipop plots displaying the correlations of THRAP3 expression with StromalScore **(A)**, ImmuneScore **(B)**, and ESTIMATEScore **(C)** in pan-cancer, respectively. **(D–F)** Pearson’s analyses of the relationships between THRAP3 expression and StromalScore **(D)**, ImmuneScore **(E)**, and ESTIMATEScore **(F)** in the indicated tumor types, respectively.

The result of the ESTIMATE analysis revealed that THRAP3 expression is negatively correlated with StromalScore in eight tumors (Figure 5A). In 17 cancer types, THRAP3 expression has a negative connection with ImmuneScore (Figure 5B). Figure 5C illustrates that THRAP3 expression is negatively connected with ESTIMATEScore in fourteen tumors. Of note, SARC has a significant negative connection to StromalScore, ImmuneScore, and ESTIMATEScore (Figures 5D–F).

### GSEA and GSVA

**FIGURE 6:**
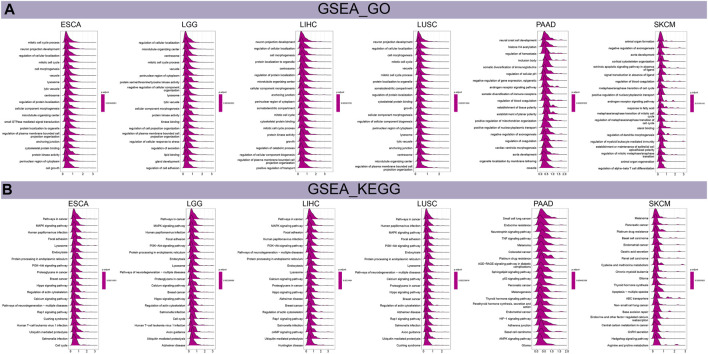
Results of GSEA. **(A)** The biological function of THRAP3 in six cancers. **(B)** KEGG pathway analysis of THRAP3 in six cancers.

We performed GSEA and GSVA to investigate the biological characteristics of THRAP3 expression in six cancers, including esophageal squamous cell carcinoma (ESCA), LGG, LIHC, LUSC, PAAD, and SKCM. Figure 6A shows that THRAP3 was predicted to be a positive regulator of cell cycle, cell growth, vacuole, lysosome, centrosome. In addition, THRAP3 is involved in regulating alpha-beta T cell differentiation. THRAP3 expression is positively connected with cancer-related pathways, including MAPK, PI3K-Akt, Hippo, calcium, Rap1, cAMP, TNF, p53, HIF-1, hedgehog pathways (Figure 6B).

**FIGURE 7:**
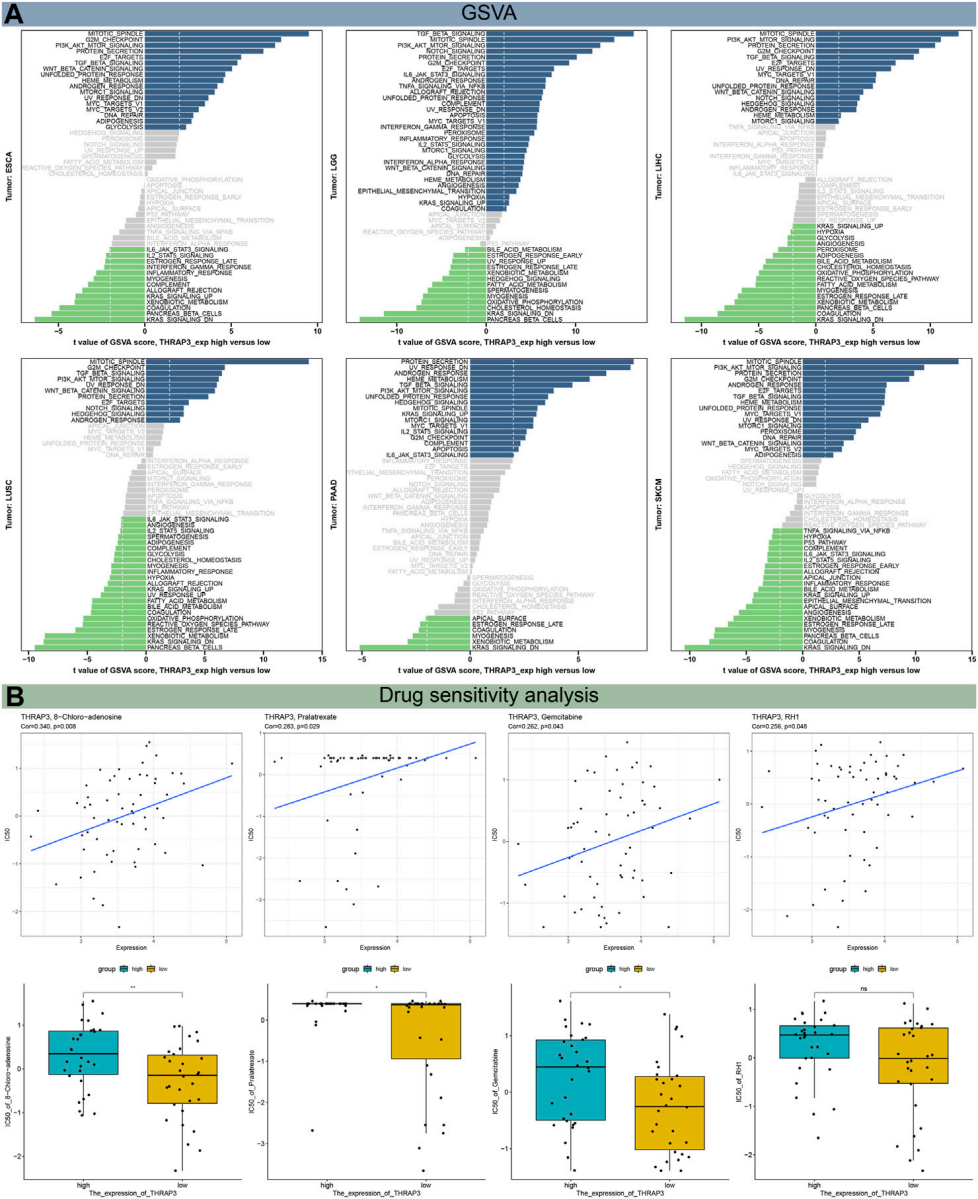
Results of GSVA **(A)** and drug sensitivity analysis **(B)**.

Results of GSVA revealed that THRAP3 was predicted to be the activator of PI3K-Akt, TGF-β, Wnt-β pathways, G2M checkpoint, MYC targets V1, V2 (Figure 7A). Additionally, THRAP3 expression negatively correlates with myogenesis, xenobiotic metabolism, coagulation, pancreas-β cells in tumor patients with THRAP3 overexpression.

### Genetic alteration analysis and drug sensitivity analysis

**FIGURE 8:**
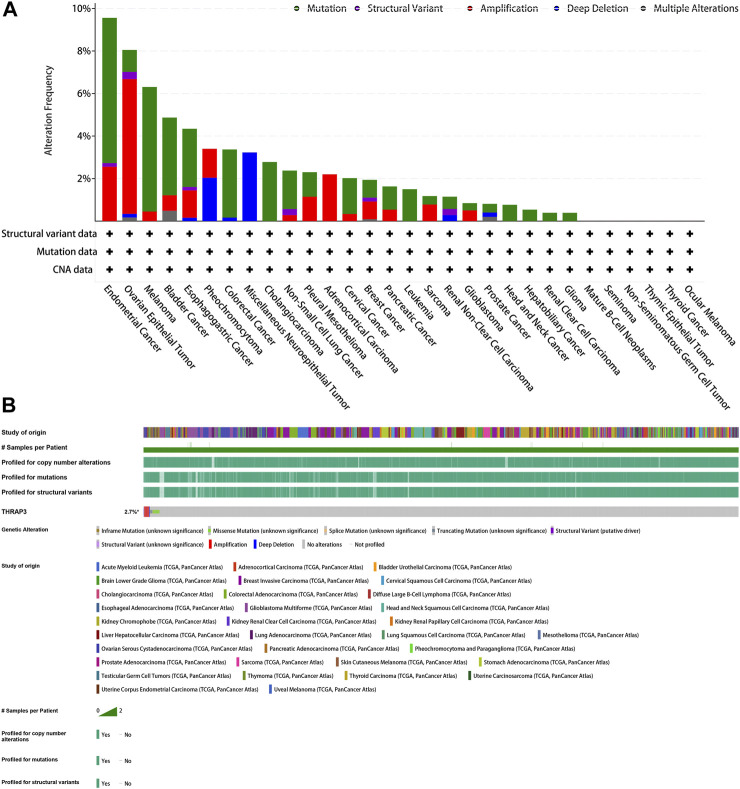
Mutation of GPC2. **(A)** Alteration frequency of GPC2. **(B)** OncoPrint visual summary of alterations in a query of GPC2 from cBioPortal.

The mutation frequency of the THRAP3 gene in endometrial cancer, ovarian epithelial cancer, and melanoma exceeds 5%. Deep deletion is the only mutation type in miscellaneous neuroepithelial tumors (Figure 8A). Figure 8B illustrates that the mutation frequency of the THRAP3 gene was 2.7% in pan-cancer. Amplification and missense mutations are the most common mutation types.

Using the CellMiner data, we conducted a drug sensitivity analysis to determine the link between THRAP3 expression and the IC50 value of target medications (Figure 7B). The result showed that THRAP3 expression is positively connected with 8−Chloro−adenosine, Pralatrexate, Gemcitabine, RH1. of note, patients with high THRAP3 expression have less sensitivity to 8−Chloro−adenosine, Pralatrexate, Gemcitabine.

### scRNA profiling of LUAD

**FIGURE 9:**
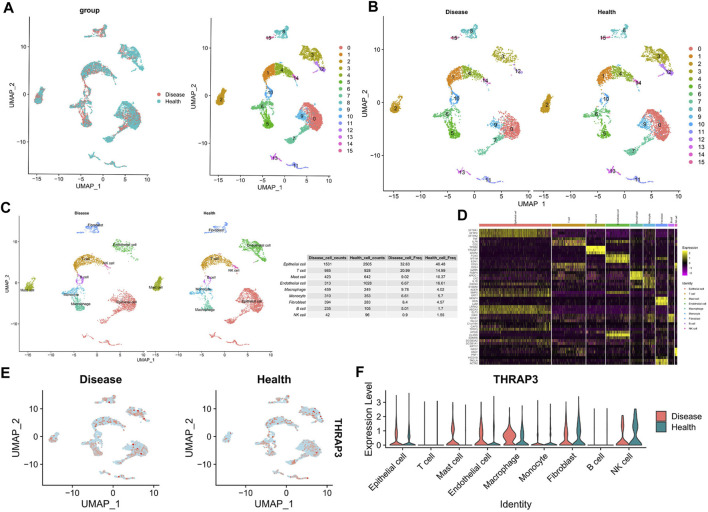
Cellular composition of the LUAD tissue microenvironment. **(A,B)** The UMAP plot shows the distribution of 16 major cell subsets. **(C)** Annotation of each cell type in LUAD tissues and adjacent normal tissues. **(D)** Heatmap shows the top 3 genes with the highest avg_logFC of each cell type. **(E)** UMAP projections showing the expression and distribution of THRAP3 in each cell type. **(F)** Violinplot of THRAP3 expression in each cell type.

Following the pre-processing of the GSE149614 dataset, we combined LUAD with adjacent healthy tissues and then performed UMAP non-linear dimension reduction. Afterward, 16 cell subclusters were formed by segregating all cells (Figures 9A, B). The “FindAllMarkers" function was used to identify DEGs in each cluster (logFC = 0.25). By utilizing the 10 subtype-specific DEGs for each cell subtype, we identified a total of 9 cell types, including epithelial cells, T cells, mast cells, endothelial cells, macrophages, monocytes, fibroblasts, B cells, and NK cells (Figure 9C). Additionally, we also evaluated the proportion of the mentioned cell types in LUAD and adjacent healthy tissues. Compared to normal tissues, LUAD tissues exhibited a higher proportion of T cells, macrophages, fibroblasts, and B cells (Figure 9D). To visualize the expression level of THRAP3 in both LUAD and normal tissues, we employed dotplots and featureplots. The results revealed that in LUAD tissues, THRAP3 is upregulated in mast cells, endothelial cells, and macrophages (Figures 9E, F).

### IHC analysis

**FIGURE 10:**
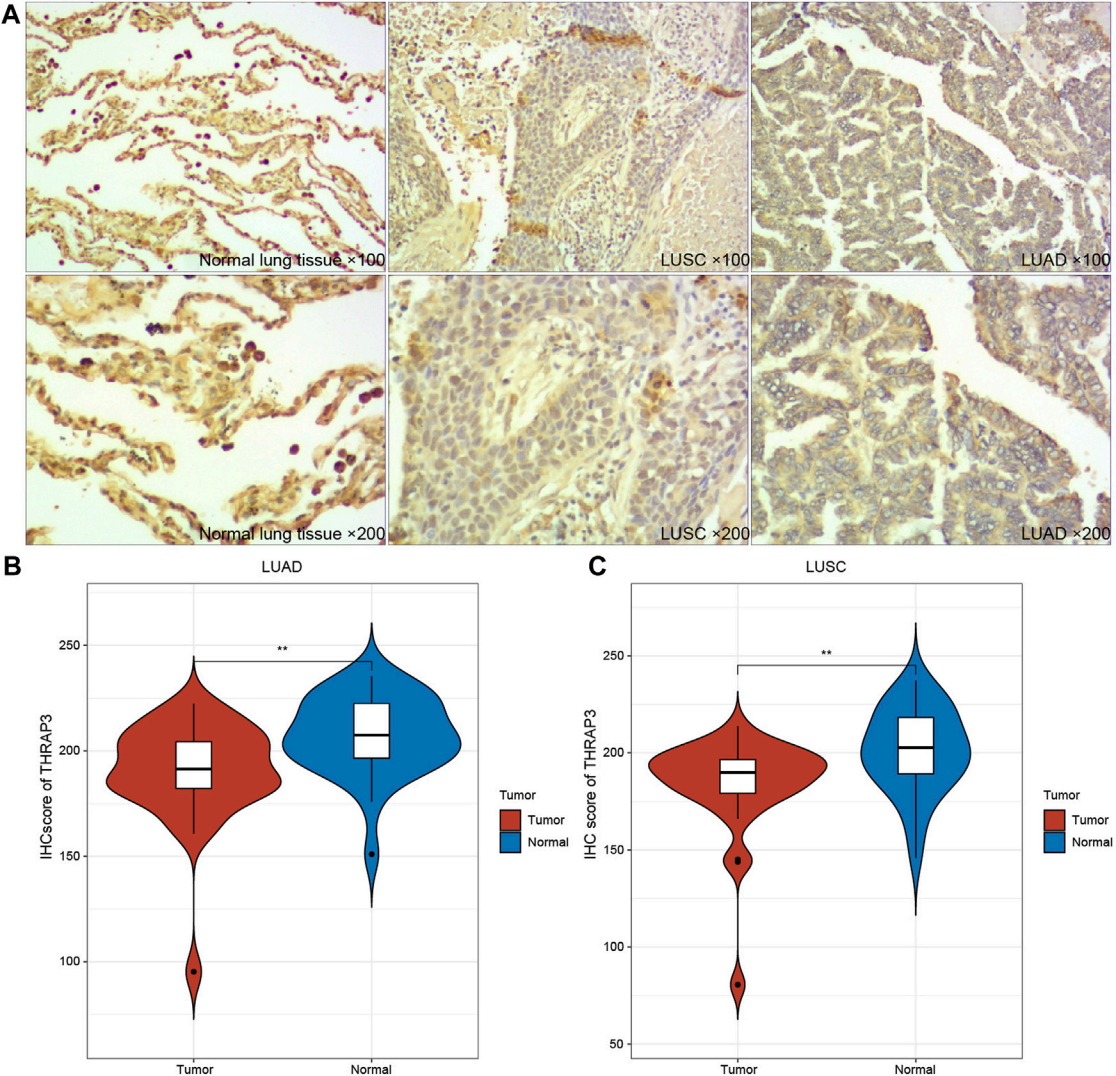
Immunohistochemical images of THRAP3 in LUSC, LUAD. **(A)** Low expression of THRAP3 in lung cancer tissues. **(B)** Differences in IHC scores between LUAD samples and normal samples. **(C)** Differences in IHC scores between LUSC samples and normal samples.

The results of IHC showed that THRAP expression levels were relatively lower in LUAD and LUSC (Figures 10A–C), compared to matched-normal tissues.

## Discussion

Recent reports suggest that THRAP3 expression is involved in pre-mRNA alternative splicing, nuclear export, and DNA damage response (Beli et al., 2012). Li et al. found that THRAP3 expression could be a potential immunotherapy target by affecting the infiltration levels of immune cells, based on the bioinformatic analysis (Li et al., 2022). In BRAF-mutated colorectal cancer, nuclear PD-L1 accelerated tumor progression by interacting with THRAP3 (Ma et al., 2022c). Thus, THRAP3 serves as a potential biomarker and immunotherapy target in various cancers, which is worth further study.

Pan-cancer analysis data revealed that THRAP3 is significantly correlated with prognosis, and TME in multiple malignancies. Of note, the IHC result suggested that THRAP3 is downregulated in LUAD and LUSC, which could be a protective gene. Thus, THRAP3 protein has the potential to act as a specific tumor biomarker.

Moreover, THRAP3 expression significantly correlates with tumor stage and age in various cancers. THRAP3 expression is higher at the early stage than at the late stage in BRCA, HNSC, KIRC, and OV. However, in patients with ACC, THRAP3 expression is lower at the early stage than at the late stage. Therefore, our data predict that ACC patients with high THRAP3 expression may have a poor prognosis. The forestplot data showed THRAP3 as a protective gene in KIRC, while in KICH, LGG, LUSC, KICH, BLCA, PAAD, ACC, and LIHC, THRAP3 is a high-risk gene. KM analysis further indicated that THRAP3 elevation had a longer survival time in KIRC. In comparison, upregulation of THRAP3 expression had a shorter survival in patients with KICH, LGG, SKCM, ACC, LGG. These results suggested that THRAP3 is a promising biomarker for tumors.

Next, we studied the relationship between THRAP3 expression and TME. The data showed that THRAP3 expression is negatively connected with StomalScore, ImmuneScore, ESTIMATEScore in most cancer types, including SARC, KIRC, LAML, LGG, PAAD, GBM, SARC, TGCT, UCEC, COAD. We hypothesize that THRAP3 plays an essential role in the formation of the TME.

Regarding the biological significance of THRAP3 expression in different cancers, THRAP3 was predicted to be a positive regulator of the lysosome, vacuole, centrosome, and cell growth, and to participate in regulating cancer-related pathways, including the PI3K-Akt, Hippo, MAPK, Rap1, calcium, hedgehog pathways. These results are worth further research.

Gene mutation analysis showed that THRAP3 gene mutations are prevalent in most cancer types. Three tumors with the highest alteration frequency of THRAP3 were endometrial cancer, ovarian epithelial cancer, and melanoma, respectively. Furthermore, tumor patients with THRAP3 overexpression have less sensitivity to 8−Chloro−adenosine, Pralatrexate, Gemcitabine based on the drug sensitivity data.

ScRNA-seq analysis revealed a significant increase in infiltration levels of T cells, macrophages, fibroblasts, and B cells in LUAD tissue compared to adjacent normal tissues, while THRAP3 was significantly upregulated in mast cells, endothelial cells, and macrophages. Our data suggests that the pathogenesis of LUAD involves alterations in both immune cell infiltration levels and THRAP3 expression.

Although we systematically analyzed the expression of THRAP3 in LUAD and evaluated the potential of THRAP3 to serve as a prognostic marker for LUAD, our study still has some limitations. Firstly, we did not thoroughly investigate the relationship between THRAP3 and immune cell infiltration. Secondly, the oncogenic role of THRAP3 in other tumors still requires further validation.

## In conclusion

Our results reveal differential expression of THRAP3 in multiple cancers relative to matched-normal tissues, and THRAP3 could be a prognostic biomarker in specific cancer types. Based on the ScRNA-seq analysis, we speculate that mast cells, endothelial cells, and macrophages may affect the progression of LUAD by regulating THRAP3 expression.

## Data Availability

The original contributions presented in the study are included in the article/Supplementary material, further inquiries can be directed to the corresponding authors.
